# Quantifying the impact of heat on human physical work capacity; part III: the impact of solar radiation varies with air temperature, humidity, and clothing coverage

**DOI:** 10.1007/s00484-021-02205-x

**Published:** 2021-10-28

**Authors:** Josh Foster, James W. Smallcombe, Simon Hodder, Ollie Jay, Andreas D. Flouris, Lars Nybo, George Havenith

**Affiliations:** 1 Environmental Ergonomics Research Centre, Loughborough University, Loughborough, LE11 3TU UK; 2 Thermal Ergonomics Laboratory, University of Sydney, Sydney, NSW Australia; 3 FAME Laboratory, University of Thessaly, Trikala, Greece; 4 Department of Nutrition, Exercise and Sports, University of Copenhagen, Copenhagen, Denmark

**Keywords:** Heat stress, Performance, WBGT, UTCI, Sunlight, Labor capacity

## Abstract

Heat stress decreases human physical work capacity (PWC), but the extent to which solar radiation (SOLAR) compounds this response is not well understood. This study empirically quantified how SOLAR impacts PWC in the heat, considering wide, but controlled, variations in air temperature, humidity, and clothing coverage. We also provide correction equations so PWC can be quantified outdoors using heat stress indices that do not ordinarily account for SOLAR (including the Heat Stress Index, Humidex, and Wet-Bulb Temperature). Fourteen young adult males (7 donning a work coverall, 7 with shorts and trainers) walked for 1 h at a fixed heart rate of 130 beats∙min^−^1, in seven combinations of air temperature (25 to 45°C) and relative humidity (20 or 80%), with and without SOLAR (800 W/m^2^ from solar lamps). Cumulative energy expenditure in the heat, relative to the work achieved in a cool reference condition, was used to determine PWC%. Skin temperature was the primary determinant of PWC in the heat. In dry climates with exposed skin (0.3 Clo), SOLAR caused PWC to decrease exponentially with rising air temperature, whereas work coveralls (0.9 Clo) negated this effect. In humid conditions, the SOLAR-induced reduction in PWC was consistent and linear across all levels of air temperature and clothing conditions. Wet-Bulb Globe Temperature and the Universal Thermal Climate Index represented SOLAR correctly and did not require a correction factor. For the Heat Stress Index, Humidex, and Wet-Bulb Temperature, correction factors are provided enabling forecasting of heat effects on work productivity.

**Supplementary Information:**

The online version contains supplementary material available at 10.1007/s00484-021-02205-x.

## Introduction

Environmental heat exposure has a negative impact on human health and physical working capacity (PWC) (Flouris et al., 2018; Foster et al., 2021a, 2021b; Ioannou et al., 2021a), incurring significant economic damage through its impact on workplace productivity (Hübler et al., 2008; Zander et al., 2015; Hsiang et al., 2017). Understanding the full effect of heat on PWC is required for economic cost and general impact analysis associated with climate change and hot weather events in general (Hsiang et al., 2017). While models of PWC based on various climate indices have recently been developed (Dunne et al., 2013; Kjellstrom et al., 2018; Foster et al., 2021b), at present, none account for the effect of solar or general thermal radiation. Given that many occupational tasks involve outdoor exposure, not accounting for solar radiation (SOLAR) is presently a significant limitation.

In 338 trials with work paced based on heart rate (limit of 130 beats·min^−1^ (moderate to heavy work)), our group recently developed empirical models for PWC based on a suite of heat stress indices (Foster et al., 2021b). However, those trials were conducted without added SOLAR. Hence, to use the equations for both conditions with and without SOLAR, correction factors may be needed, especially for heat stress indices that do not intrinsically account for this parameter. For example, although Wet-Bulb Temperature (*T*_wb_), Humidex, and Heat Index strongly predict PWC in shaded environments (Foster et al., 2021b), they are calculated from air temperature (*T*_a_) and relative humidity (RH) alone, and therefore cannot accommodate conditions in which there is additional SOLAR. Moreover, while wet-bulb globe temperature (WBGT) and universal thermal climate index (UTCI) account for radiation in their calculation (Havenith and Fiala, 2015), their correct sensitivity to radiation must also be validated empirically. For WBGT and UTCI, one equation linking PWC to the thermal climate would be expected since they account for radiation, whereas correction factors may be required for *T*_wb_, Humidex, and Heat Index.

SOLAR radiation is electromagnetic radiation emitted from sunlight. SOLAR radiation covers a wide spectrum (~280 up to 780 nm), with the short (visible light covers 400–780 nm) and longer wave (near infrared at 780 nm) components transferring energy. Primarily, it is the total energy transferred (i.e., global intensity) that determines the impact on the human rather than the wavelength (Hodder and Parsons, 2007, 2008). The wavelength does, however, influence the absorbed energy, where e.g. clothing color/albedo affects the absorption in the visible spectrum (Havenith et al., 2006a, 2006b, 2006c) but not in the long wave range (Bröde et al., 2008, 2010). When SOLAR radiation is absorbed and retained by the human body, it increases body heat content which elevates thermal and cardiovascular strain (Bröde et al., 2008, 2010; Havenith et al. 2006b). Studies involving outdoor activity in the sun show marked elevations in thermal strain compared with shaded conditions, for the same *T*_a_ (Adolph, 1947; Hardy and Stoll, 1954; Nielsen et al., 1988; Gonzalez et al., 2012; Otani et al., 2017, 2019). The detrimental effects of solar radiation on thermal strain (Stolwijk and Hardy, 1965; Gagge and Hardy, 1967; Nielsen, 1990; Havenith et al. 2006b), maximal exercise performance (Otani et al., 2016), cognitive function (Piil et al., 2020), and thermal comfort (Hodder and Parsons, 2007) have also been investigated with SOLAR simulation lamps, a source of artificial, non-ionizing radiation with total energy intensity up to 1 kW·m^−2^.

Given the interaction between SOLAR and the level of human heat strain, exposure to sunlight during physical work is likely to reduce PWC. However, the extent to which SOLAR influences PWC across wide variations of *T*_a_ and relative humidity (RH) is unknown. For example, the impact of SOLAR on PWC may not fully be explained by the pure physics of SOLAR heat gain, but instead due to an interaction with human physiology. Such an interaction may depend on the atmospheric environment (*T*_a_ and RH combination) or, presuming a fixed solar load, may be consistent regardless of the environment. Moreover, it is unknown to what extent protective clothing alters this response. While clothing color and fabric can have a significant impact on overall solar heat gain (Nielsen, 1990; Havenith et al., 2006a, 2006b, 2006c; Ioannou et al., 2021b), direct comparisons between exposed and clothed skin are unavailable. The above knowledge is essential for accurate PWC forecasting, and to determine what combinations of *T*_a_ and RH should be prioritized with shading solutions and clothing modifications.

The primary aim of the present study was to quantify the impact of SOLAR on PWC, based on clothing coverage (low or high), across a wide range of temperature and relative humidity combinations (*T*_a_=25–45°C; RH=20–80%). The secondary aim was to generate correction factors so PWC can be modeled outdoors using heat stress indices that do not ordinarily account for SOLAR in their calculation (namely, *T*_wb_, Humidex, and Heat Index). The final aim was to test the sensitivity of WBGT and UTCI to added solar heat loads, confirming whether our earlier published functions (Foster et al., 2021b) can be used for indoor and outdoor work settings.

## Methodology

### Overview

Participants were randomly allocated into either a low or high clothing coverage group (*between participant*), and subsequent *within participant* comparisons (i.e., SOLAR vs. SHADE) took place in different *T*_a_ and RH combinations. We adopted a physical work protocol in a climate chamber which simulates the self-pacing behaviors adopted in the field. We refer the reader to our companion paper (Foster et al., 2021b) for a thorough rationale and description of the protocol. A study schematic is shown in Fig. S1 (supplementary file).

### Ethical approval

This study was approved by the Loughborough University Ethics Committee and was performed in-line with the Declaration of Helsinki. Participants were provided with an information sheet that detailed the risks and requirements of the experiment before providing written informed consent. Participants conducted a health screening questionnaire prior to the start of the experiments.

### Location and timeline

The data collection took place in custom-made environmental chambers (TISS performance chambers, UK) located within the Environmental Ergonomics Research Centre, Loughborough University. Data collection ran from September 2018 to May 2019.

## Participants

A total of 13 participants took part in the study. The total number of trials performed varied between participants, ranging from 4 to 14 (median = 10). Table 1 displays participant characteristics for each experimental group. To reduce any impact of heat acclimation throughout the trial period, the number of experiments were capped at three per week, but seldom exceeded two per week. The fixed heart rate approach also limits any sustained rises in body temperature that are required on a daily basis to elicit a heat acclimated phenotype (Fox et al., 1963; Foster et al., 2021b). Moreover, participants were not permitted to take part in the study if they recently visited a hot climate.

**Table 1:** Participant characteristics

Variable	Low clothing coverage (*n* = 7)		High clothing coverage (*n* = 7)	
Age (years)	25 ± 3	(20–29)	23 ± 3	(20–28)
Height (cm)	177 ± 5	(171–185)	178 ± 6	(172–192)
Mass (kg)	74 ± 10	(59–92)	74 ± 11	(59–94)
BSA (m^−2^)	1.9 ± 0.1	(1.7–2.2)	1.9 ± 0.1	(1.7–2.1)
BMI (kg∙m^−2^)	23 ± 2	(20–28)	23 ± 3	(20–29)
Body fat (%)	18 ± 6	(10–27)	14 ± 4	(9–21)
***V̇***O_2max_ (L·min^−1^)	3.8 ± 0.8	(2.6–5.9)	4.2 ± 0.0	(2.7–5.3)
***V̇***O_2max_ (mL·kg^−1^·min^−1^)	52 ± 9	(40–65)	56 ± 5	(44–64)

Data are presented as means ± SD. The data ranges are presented in parentheses

*V̇*O_2max_, maximal oxygen consumption; BMI, body mass index; BSA, body surface area

### Experimental design

Thirteen young adult male participants were allocated to either a low or high clothing coverage group. One participant completed trials in both clothing conditions, totaling *n*=7 in each. On visit 1, participants completed a body composition assessment and graded exercise test on a treadmill to determine maximal oxygen consumption (*V̇*O_2max_). See “Preliminary trial” section for details on these tests. On visit 2, participants completed an experimental trial in a reference/control condition at 15°C, 50% RH. On subsequent visits (all trials on separate days), participants completed experimental trials in up to seven different combinations of *T*_a_ and RH. In terms of the *T*_a_ and RH combination, trials were completed in a randomized order, but a SOLAR versus SHADE comparison trial was always completed sequentially (i.e., one followed the other, although always on separate days). During each experimental trial, participants walked on a treadmill for up to 60 min at a fixed heart rate of 130 beats·min^−1^. A total of 66 and 62 hot trials were completed in low and high clothing coverage, respectively. In the supplementary file, Table S1 displays the number of trials performed by each participant according to the air temperature and relative humidity combination. A study schematic is shown in the supplementary file (Fig. S1).

### Experimental controls

Participants completed experimental sessions at the same time of day to minimize the potential effect of circadian rhythm on outcome variables (Waterhouse et al., 2004). However, it is worth noting that, apart from changes in absolute core temperature (*T*_core_), physiological effector responses are unaffected by time of day (Ravanelli and Jay, 2020). Participants presented to the laboratory in a hydrated state (confirmed by urinary analysis) and refrained from caffeine 12 h prior to each trial. Finally, participants were asked to refrain from alcohol and vigorous exercise 24 h before each trial.

### Preliminary trial (visit 1)

The preliminary visit involved an anthropometric assessment and a submaximal (walking) test of maximal oxygen consumption (*V̇*O_2max_) performed on a treadmill. At a fixed walking speed of 4.5 km/h, the submaximal test followed a ramp protocol in which the gradient increased by 5% every 3 min until a steady state heart rate of 85% age-predicted maximum was attained. Combined with indirect calorimetry to continuously assess *V̇*O_2_ uptake, *V̇*O_2max_ was predicted by extrapolating *V̇*O_2_ to age-predicted maximum heart rate. The protocol is set out in more detail in our companion paper (Foster et al., 2021b). Body composition was assessed using a Tanita scale (MC-780MA; TANITA Corporation, Japan) while participants were dressed in underwear only.

### Experimental protocol

Upon arrival, participants inserted a rectal thermistor (VIAMED, Yorkshire, UK) to a depth of 10 cm past the anal sphincter to monitor internal (rectal) temperature, which allowed continuous monitoring of *T*_core_ throughout each trial. Participants subsequently voided their bladder and provided a urine sample which was used for the assessment of urine specific gravity. If urine specific gravity exceeded 1.020, participants drank 500 ml of water and provided another sample after 20 min (Armstrong et al., 1994). Skin thermistors (Grant Instruments Ltd, Corby, UK) were then placed onto each participant at six sites (the upper back, lower back, chest, arm (triceps), thigh (quadriceps), and calf) with a breathable, hypafix tape (BSN medical, D-22771, Hamburg, Germany). The mean skin temperature (*T*_skin_) was calculated by adapting the Ramanathan equation (1964). The original equation is equal to

1 $$\boldsymbol{Original}\ {\boldsymbol{T}}_{\boldsymbol{skin}}=0.3{T}_{chest}+0.3{T}_{arm}+0.2{T}_{thigh}+0.2{T}_{thigh}\ \left[{}^{\circ}\boldsymbol{C}\right]$$

To integrate additional *T*_skin_ sites in the radiated area at the upper and lower back, *T*_chest_ was replaced by *T*_torso_, providing equal weight to the front and back temperatures of the torso, as below:

2 $$\boldsymbol{Adapted}\ {\boldsymbol{T}}_{\boldsymbol{skin}}=0.3{T}_{torso}+0.3{T}_{arm}+0.2{T}_{thigh}+0.2{T}_{thigh}\ \left[{}^{\circ}\mathbf{C}\right]$$

where *T*_torso_ was calculated by

3 $${\boldsymbol{T}}_{\boldsymbol{torso}}=0.15{T}_{chest}+0.075{T}_{upperback}+0.075{T}_{lowerback}\ \left[{}^{\circ}\boldsymbol{C}\right]$$

The value for mean *T*_skin_ was reported as the average score of “adapted *T*_skin_” during a 1-h trial. Temporal *T*_skin_ and *T*_core_ responses are shown in supplemental Fig. S4 for each condition, presented as the average score at each time point, for each condition.

### Physical work simulation

The treadmill was programmed to control workload to achieve the desired heart rate of 130 beats·min^−1^. The treadmill speed and grade were never manually controlled by the researchers or participants. The treadmill incline did not change until the speed reached its maximum of 6 km·h^−1^. Thereafter, the speed did not change unless the incline fell back to zero. Each test was set to last a maximum 1 h, but in more extreme heat the speed often fell to zero before this time. If speed fell to zero (i.e., resting heart rate is ≥130 beats·min^−1^ ) the participants exited the chamber and no more exercise took place.

### Calculation of percentage physical work capacity

A predictive equation based on treadmill speed and grade (Ludlow and Weyand, 2017) was used to calculate total cumulative energy generated in the present study. The original equation was expanded to convert energy generated in *V̇*O_2_ to total kilojoules. This equation and its validation based on 365 expired air samples are available in our companion paper (Foster et al., 2021b).

### Clothing

Participants were separated into two clothing groups before undertaking trials with and without SOLAR radiation. See supplementary file for full list of *n* numbers in each condition. The two conditions were chosen to provide minimal/low or high clothing coverage of the skin. In the low clothing trials, subjects wore underwear, standardized shorts, socks, and trainers. In the high clothing trials, subjects wore the same as the low clothing trial, with the addition of a standardized cotton t-shirt, and a standardized full body protective coverall (65% polyester, 35% cotton). The intrinsic clothing insulation of the low and high coverage ensembles were estimated as 0.04 and 0.133 m^−2^·K·W^−1^ (0.26 and 0.86 Clo), respectively, based on the International Standard (ISO9920, 2009). The evaporative resistance was estimated at 0.007 and 0.024 m^−2^·kPa·W^−1^ for the low and high clothing conditions, respectively (ISO9920, 2009).

### Environmental logging

A Quest-temp model 34 meter was used to record wet-bulb globe temperature (WBGT) at 1-min intervals. The approach taken to measure WBGT is described below. A Testo model 435-2 with hot-wire probe was used to record *T*_a_ (non-solar), RH, and air velocity and logged at 1-min intervals.

### Calculation of heat stress indices

#### Wet-bulb globe temperature (WBGT)

For all non-solar/shade trials, the average value for dry bulb, wet bulb, and globe temperature were used to assess WBGT (WBGT= 0.7*T*_wb_ + 0.2*T*_globe_ + 0.1*T*_a_) for a given work bout. The WBGT monitor was placed on a meter-high stand within ~0.5 m of the participants.

For the SOLAR trials, a WBGT assessment of the environment was undertaken on a separate day, for each temperature and humidity combination (seven environments total). Live WBGT measurement during the trials was not practical because the WBGT meter needed to be placed in the same location as the participant to measure the heat load projected onto the participant. Due to the specific directionality of each SOLAR lamp, the radiation intensity was not evenly distributed throughout the environmental chamber. Hence, we assessed WBGT placed where the participants would be standing during trials, to yield an accurate assessment. SOLAR intensity also varied with height, so WBGT was measured at four different heights (the average of all four was used). For example, globe temperature was ~5°C lower at 1.6 m height (517 ± 24 W·m^−2^) compared with that at 0.7 m height (1065 ± 36 W·m^−2^), independent of air temperature and humidity. The dry bulb was shielded from radiation using a foil-coated cardboard plate, with the sensor upwind from the shield. The same WBGT value was used for every participant in each SOLAR condition.

#### Psychrometric wet bulb temperature (T_wb_)

Psychrometric wet bulb temperature was calculated based on Bernard and Pourmoghani (1999):

4 $${{T}}_{{wb}}=0.376+5.79{{P}}_a+\left(0.388-0.0465{P}_{{a}}\right){{T}}_{db}\ \left[{}^{\circ}C\right]$$

where *P*_a_ is the ambient water vapor pressure (measured in kPa) and *T*_db_ is the dry bulb temperature (air temperature). *P*_a_ was calculated using the following equation (Parsons, 2010):

5 $${P}_a={e}^{\left(18.956-4030.18{T}_a+235\right)}\times \frac{Rh}{100}\left[{kPa}\right]$$

where *T*_a_ is ambient temperature (°C) and RH is relative humidity (0–100).

### Universal Thermal Climate Index (UTCI)

UTCI was calculated for each condition in Excel (www.climatechip.org/excel-wbgt-calculator), which uses a six-parameter polynomial model (Bröde et al., 2012). *T*_a_, RH, globe temperature, and air velocity were used for the calculation. For globe temperature, the mean of the four heights (0.23, 0.69, 1.15, and 1.61 m) described previously was used. The measured air velocity at participant level was 0.32 m·s^−1^ (average for all trials). The air velocity was imputed as 0.5 m·s^−1^ (approximate value when converted to 10 m above ground level to provide the relevant meteorological wind speed).

### Humidex

The Humidex was computed as below (Masterton and Richardson, 1979; Rana et al., 2013):

6 $$Humidex={T}_a+\frac{5}{9}\left(\left[6.112\times {10}^{\left(\frac{7.5{T}_a}{237.7+{T}_a}\right)}\times \frac{Rh}{100}\right]-10\right)$$

where *T*_a_ is air temperature (°C) and *RH* is relative humidity in percent (0–100).

### Heat Index

The Heat Index was computed as below (Rothfusz, 1990):

7 $$Heat\ Index=-42.379+2.04901523{T}_{{a}}+10.14333127 Rh-0.22475541{T}_{{a}}\bullet Rh-6.83783\times {10}^{-3}{T}_{{a}}^2-5.481717\times {10}^{-2}R{h}^2+1.22874\times {10}^{-3}{T}_{{a}}^2\bullet Rh+8.5282\times {10}^{-4}{T}_{{a}}\bullet R{h}^2-1.99\times {10}^{-6}{T}_{{a}}^2\bullet R{h}^2\ \left[{}^{\circ}{F}\right]$$

where *T*_a_ is in degrees Fahrenheit and RH is 0–100. The Heat Index in Fahrenheit was subsequently converted to Celsius.

### SOLAR simulation lamps

SOLAR was produced artificially using compact source iodide lamps (CSI; Thorn Lighting, Durham, UK) (Beeson, 1978). The lamps filter out ionizing radiation to negligible values and thereafter produce a similar spectral content to that of sunlight. An array of three vertically aligned 1000-W metal halide lamps were placed 2.3 m posterior to the participant. Although naturally, the sun would project radiation at an angle above 0°, we chose to keep the altitude and azimuth at 0° to (1) reduce the confounding effect of reflected radiation from the ground back to the participant (therefore measuring *direct* radiation only) and (2) to simulate a worst case scenario condition with respect to the percent skin surface area exposed to radiation. By maximizing the projected area in our work, it allows users of our proposed models to interpolate the effects of SOLAR at ranges of projected area observed in natural environments. In the SOLAR trials, the lamps were switched on at least 1 h before activity to allow stabilization. The average intensity of radiation across all exposed body regions for all trials was 807 ± 24 W/m^−2^, as measured immediately before and after each experiment (CM11 Pyranometer; Kipp and Zonen, The Netherlands). The pyranometer was pointed at the lamps at a distance equivalent to where the participant would stand, at the four heights mentioned previously (0.23, 0.69, 1.15, and 1.61 m). This level of SOLAR is typical for what is observed under a clear sky during the hottest part of the day, as has been shown in the USA (Xia et al., 2017), Cyprus (Ioannou et al., 2017), and Great Britain (Monteith and Unsworth, 1990). The projected area (*A*_p_) was estimated at 24.2% total body surface area, based on a SOLAR altitude and azimuth of 0° (Underwood and Ward, 1966). The average body surface area was 1.90 m^2^ (range 1.69 to 2.21 m^2^), resulting in a total radiant heat load of 382 W (range 339 to 441 W).

Statistical analysis

Statistical analyses were conducted using IBM SPSS version 27 and GraphPad Prism version 8. In SPSS, a linear mixed model with fixed (condition (SOLAR or SHADE), temperature (three or four levels depending on humidity), and clothing (low or high coverage)) and random (subject ID) effects was used to compare physical work capacity (PWC%) responses between SHADE and SOLAR trials. Four levels of temperature were assessed in dry climates (25, 35, 40, and 45°C), and three were assessed in humid climates (25, 30, and 35°C). Data are reported as mean difference ± SE, 95% CIs of the difference, and effect size. Effect size was calculated as

8 $$\mathbf{Effect}\ \mathbf{size}=\frac{{Mean}\ {difference}}{{SD}\ }$$

where mean difference is the group average difference between PWC in SHADE versus SOLAR, and SD is the standard deviation of the differences. The threshold values of 0.2, 0.5, and 0.8 were used to indicate a small, moderate, and large effect, respectively (Cohen, 1988).

In accordance with our companion paper (Foster et al., 2021b), we used a sigmoidal expression to determine the overall impact of heat on PWC%. All modeling was performed using GraphPad Prism version 8. The model takes the form

9 $$\boldsymbol{Physical}\ \boldsymbol{Work}\ \boldsymbol{Capacity}\%=\frac{100}{1+{\left(\frac{PWC50}{x}\right)}^{HillSlope}}$$

where *x* represents the predictor studied (e.g., WBGT), *PWC50* is the value of *x* that elicits 50% PWC, and *HillSlope* defines the steepness of the curve. The *HillSlope* and *PWC50* parameters were calculated from the GraphPad Prism analysis to find the optimal fit to the data (producing the least variance). The extra sum of squares *F* test was used to determine if best fit values of selected parameters (PWC50 and *HillSlope*) differed significantly between SHADE and SOLAR datasets (Turner et al., 2015), i.e., is the error in the model reduced using specific parameters for each dataset versus using global/shared parameters. The alpha value for all significance testing was set as *p* <0.05.

If a separate model was required for inclusion of the SOLAR data (based on a significant *F* test), correction factors were generated based on the difference in PWC% between the SHADE and SOLAR models. We then modeled the *difference* in PWC between the SHADE and SOLAR models to form correction factors based on the solar intensity. The correction factors were then used to estimate PWC during sunlight exposure, for heat indices that do not intrinsically account for radiation in their calculation (i.e., *T*_wb_, Humidex, and Heat Index). The correction factor was formed based on the following template:

10 $$\boldsymbol{Physical}\ \boldsymbol{Work}\ \boldsymbol{Capacity}\%= PW{C}_{{shade}}-\left(\frac{\alpha }{800}\bullet \frac{A_p}{24.2}\bullet Sin\theta \bullet PW{C}_{{corr}}\right)$$

where *PWC*_shade_ calculates percent PWC in shaded conditions (use equations set out in Foster et al., 2021b), *PWC*_corr_ is the correction factor observed in this experiment, and $\frac{\alpha }{800}\bullet \frac{A_p}{24.2}\bullet Sin\theta$ is a linear scaling factor which interpolates the SOLAR impact based on the actual present radiation level. Equations for *PWC*_shade_ are available in our companion paper (Foster et al., 2021b); *α* is the SOLAR intensity (W∙m^−2^). In addition, 800 W∙m^−2^ was the reference for the radiation level used in the present experiment. Using a SOLAR intensity of greater than 800 W∙m^−2^ is possible but extrapolates beyond our empirical dataset rather than interpolating; *A*_p_ is the projected area (the surface area of skin exposed to radiation), expressed as a percentage of total body surface area. Moreover, 24.2% was used in the present study and represents a worst-case scenario (i.e., maximum possible surface area exposed to SOLAR). Sin*θ* is the sine of the angle (*θ* in degrees) between the SOLAR beam and the surface onto which it projects. Sin*θ* values range from 0 to 1. Values for *A*_p_ can be calculated based on SOLAR altitude and azimuth (Underwood and Ward, 1966).

The predictive power of core and mean *T*_skin_ for estimating PWC% was assessed using the same function as described in Eq. (9), with the *x* value representing core or *T*_skin_. The relationship between these two thermometric variables and WBGT were assessed with a basic linear model.

## Results

### Physical work capacity

#### Dry climate

**Fig. 1:**
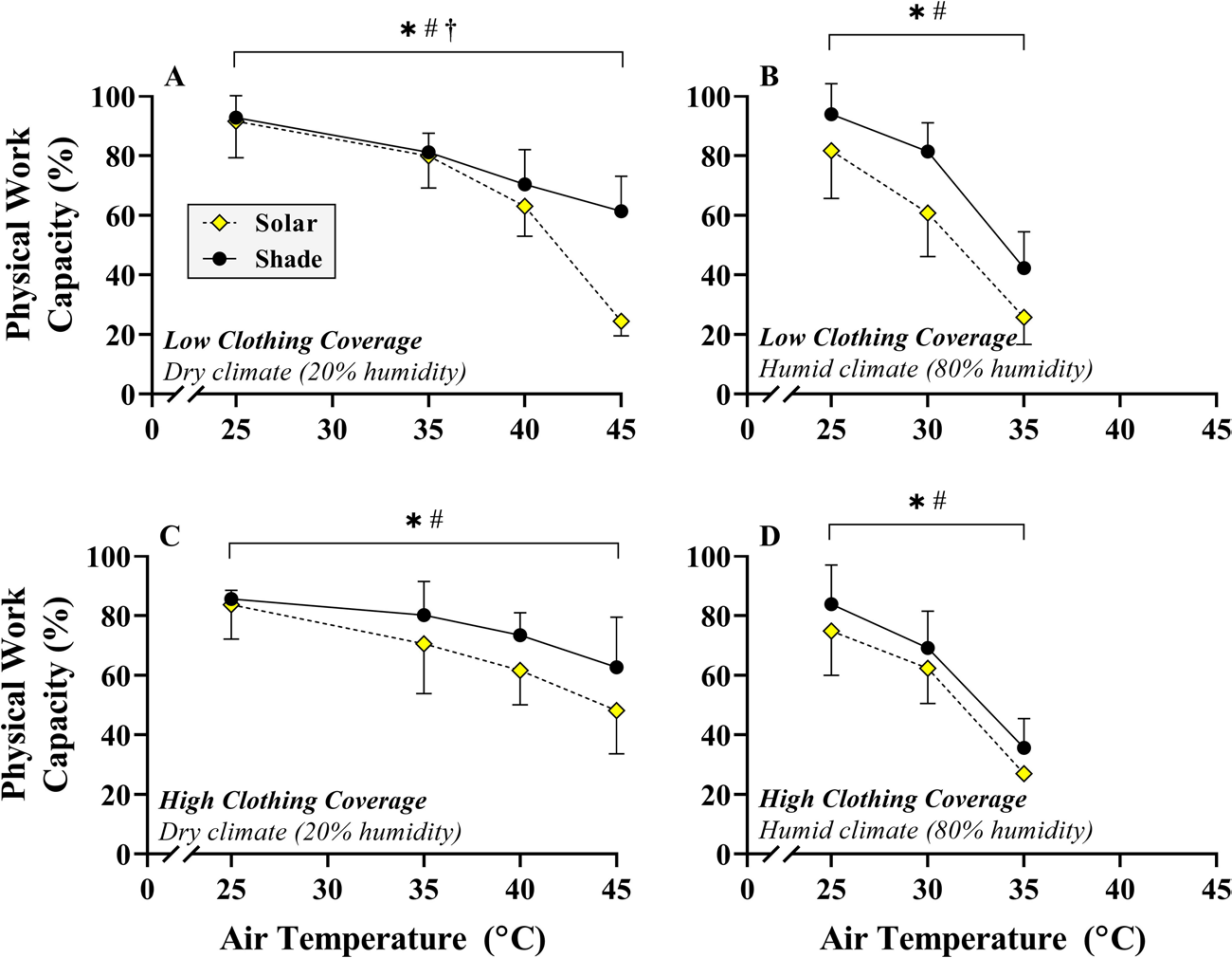
Change in absolute physical work capacity in SHADE (black circles) with SOLAR (yellow diamonds) in low (**A** and **B**) or high (**C** and **D**) clothing coverage conditions, in dry (**A** and **C**) and humid (**B** and **D**) climates. **✱ #** denote main effects for condition and air temperature, respectively. **†** denotes an interaction effect between condition and air temperature

For absolute PWC (%), significant main effects were found (Fig. 1A, C) for condition (SOLAR vs. SHADED) and air temperature (*T*_a_) (*p* < 0.05), but not for clothing (*p* > 0.05). There was a significant three-way interaction term, indicating that the impact of SOLAR on PWC varied with air temperature and clothing (*p* < 0.05). In low clothing coverage, there was no effect of SOLAR on PWC at 25°C (ΔPWC =−1 ± 13%, effect size (ES) = 0.09) or 35°C *T*_a_ (−1 ± 9%, ES = 0.13). However, SOLAR decreased PWC at 40°C (−7 ± 5%, ES = 1.32) and 45°C *T*_a_ (−37 ± 7%, ES = 5.23). In high clothing coverage, there was no significant effect of SOLAR on PWC at 25°C *T*_a_ (−2 ± 13%, ES = 0.14). However, SOLAR reduced PWC at 35°C (−6 ± 10%, ES = 0.57), 40°C (−12 ± 8%, ES = 1.57), and 45°C *T*_a_ (−15 ± 12%, ES = 1.29).

#### Humid climate

Main effects were found for condition (SOLAR vs. SHADE) and *T*_a_ (*p* < 0.05), but not for clothing (*p* > 0.05) (Fig. 1B, D). There were no significant interaction terms, indicating that the impact of SOLAR on PWC was not dependent on the air temperature or clothing (*p* > 0.05). In low clothing coverage, SOLAR decreased PWC at 25°C (−12 ± 13%, ES = 0.95), 30°C (−20 ± 15%, ES = 1.39), and 35°C *T*_a_ (−15 ± 3%, ES = 4.96). In high clothing coverage, SOLAR reduced PWC at 25°C (−9 ± 9%, ES = 1.03), 30°C (−7 ± 7%, ES = 1.03), and 35°C *T*_a_ (−9 ± 8%, ES = 1.11).

The individual level PWC responses in each condition are available in the supplementary material (Figs. S2 and S3).

Role of skin and core temperature in predicting physical work capacity

**Fig. 2:**
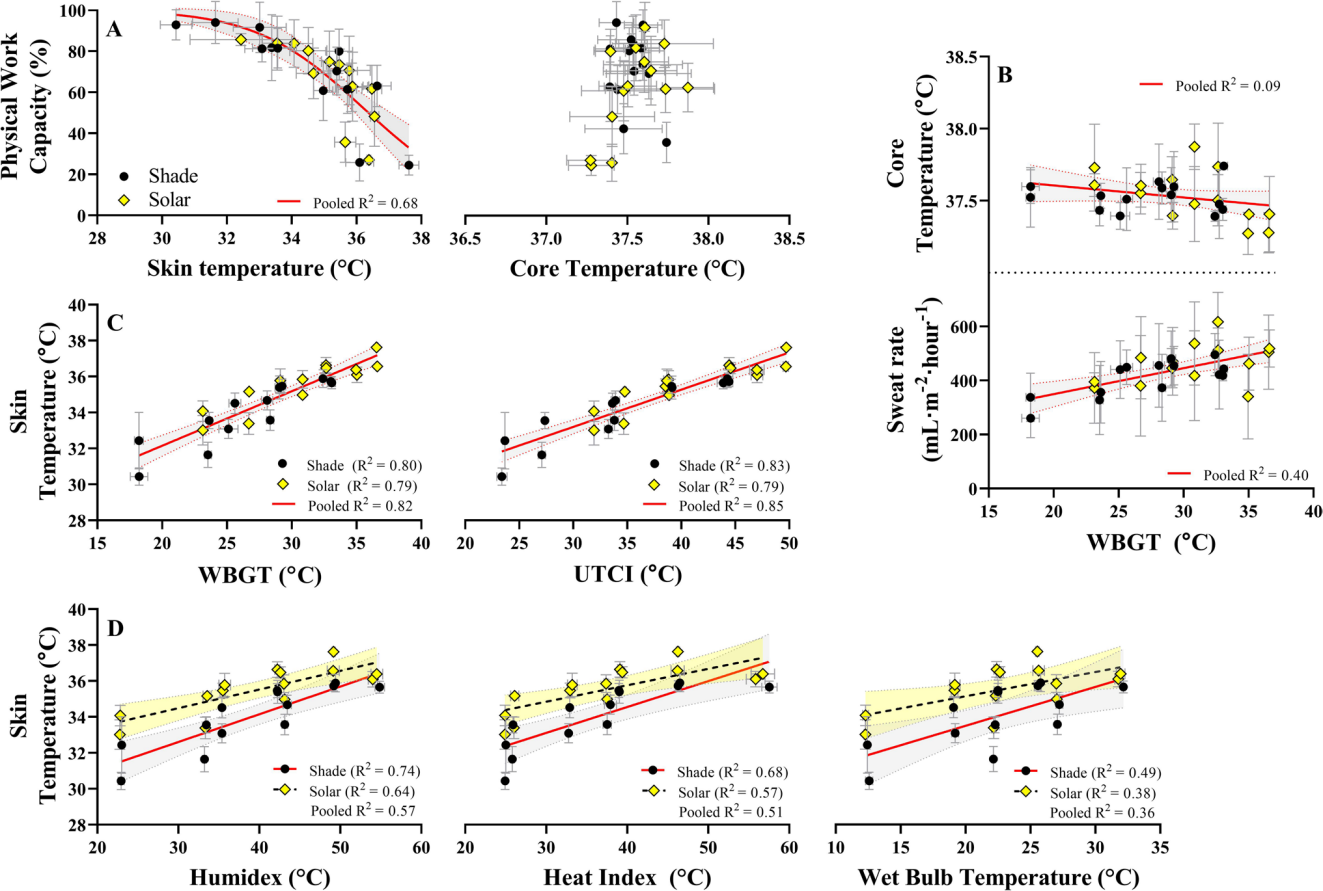
**A** The predictive value of average skin and core temperature on physical work capacity in the heat. Skin temperature was a strong predictor of physical work capacity (*R*^2^ = 0.68), whereas core temperature had no predictive value. **B** Average core temperature and sweat rate response in each trial. There was no relationship with core temperature and WBGT since work output decreases as WBGT increases. The group average sweat rate showed a modest increase with WBGT. **C** Average skin temperature plotted against WBGT and UTCI. Data for SOLAR and SHADE can share parameter values for intercept and slope, resulting in a single regression line. **D** Average skin temperature response plotted against Humidex, Heat Index, and Wet Bulb Temperature. Data for SOLAR and SHADE cannot share parameter values since these heat stress indices do not account for solar radiation. Therefore, separate regression lines are required for each dataset

Figure 2A shows the independent effect of *T*_skin_ and *T*_core_ (average of each trial) on PWC. During physical work, we show that *T*_skin_ (but not *T*_core_) is a strong predictor of PWC in the heat. While SOLAR results in a higher *T*_skin_ for the same *T*_a_ and RH, the predictive power of *T*_skin_ for estimating PWC does not appear to be impacted by addition of SOLAR, i.e., the same *T*_skin_/PWC relation holds true for both SOLAR and non-SOLAR conditions (*R*^2^ = 0.69). Using WBGT as an example heat stress metric, Fig. 2B demonstrates that *T*_core_ did not change as a function of environmental heat load during our self-paced work simulation. Figure 2C and D shows the *T*_skin_ response to increased environmental heat stress, based on WBGT and UTCI. WBGT and UTCI were strong predictors of *T*_skin_, and the strength of the prediction improved with the inclusion of SOLAR data. Analysis demonstrated that for WBGT and UTCI, separate values for *intercept* and *slope* parameters are not required for the SHADE and SOLAR datasets (*p* > 0.05). In contrast, separate parameter values *are* required for predicting *T*_skin_ based on Humidex, Heat Index, and *T*_wb_ (*p* < 0.05) as the data separate in a SOLAR and a SHADE line. For predicting *T*_skin_ based on environmental heat, we recommend using the equations provided in our recently published study using a larger dataset (see supplementary table S3 in Foster et al., 2021b). The primary aim of this analysis presented in Fig. 2C and D was to determine whether the heat stress indices appropriately reflect the SOLAR effect, which for the indices in 2C was not the case.

### Modeling physical work capacity with solar radiation

**Fig. 3:**
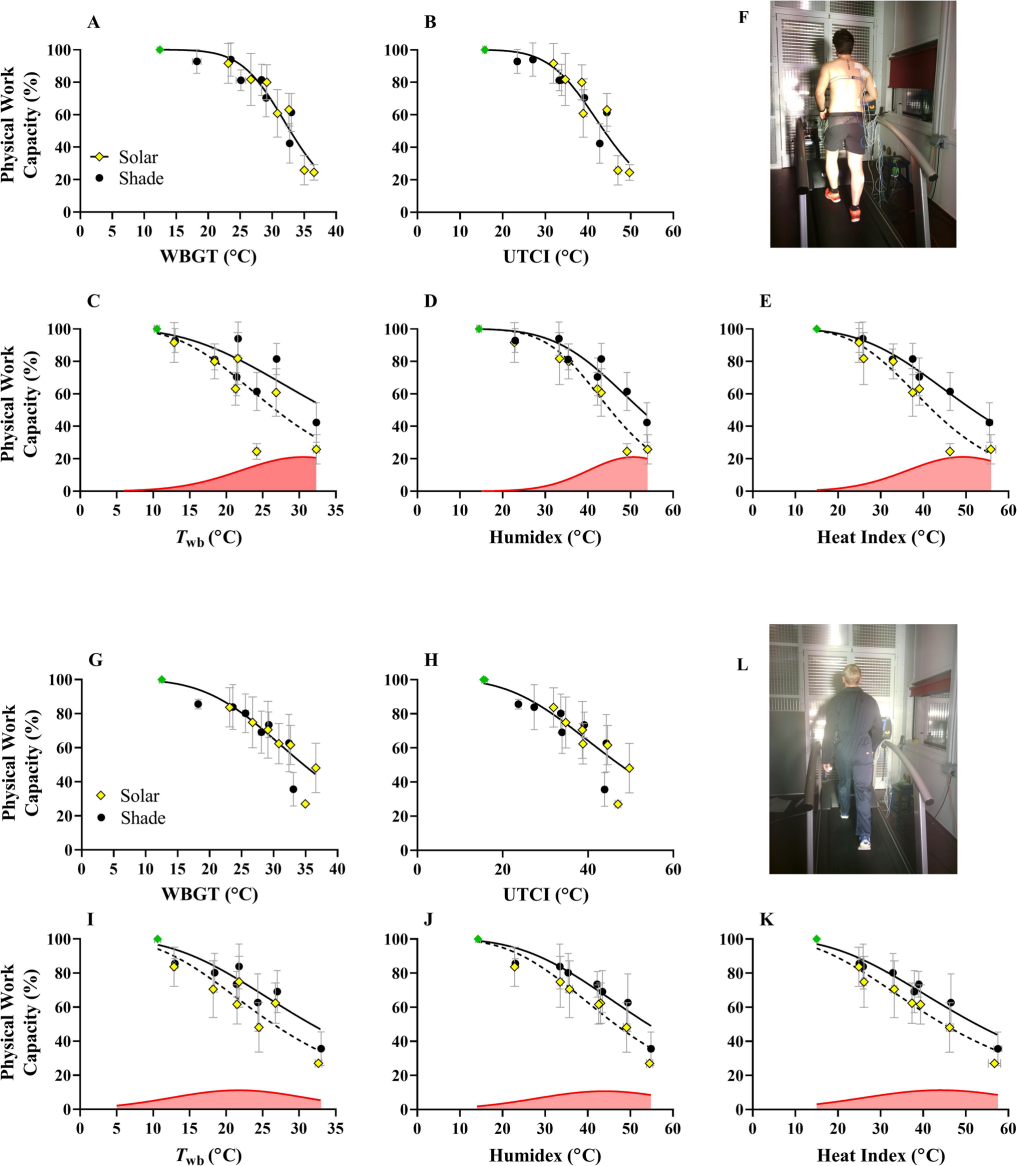
Physical work capacity (PWC) with low (**A**–**F**) and high clothing coverage (**G**–**L**). Black circles and yellow diamonds represent SHADE and SOLAR trials, respectively. For WBGT and UTCI, a pooled model is sufficient for predicting PWC even if solar data are included. For psychrometric wet-bulb (*T*_wb_), Humidex, and Heat Index, separate models are required if predicting PWC during SOLAR exposure. This was true for low and high clothing coverage. Here, the solid line and dotted line in figures represent SHADE and SOLAR data models, respectively. The *difference* in PWC (i.e., SHADE − SOLAR) for models followed a Gaussian distribution and is shown by the red area fill. These Gaussian models are shown in Table 4 and were required for the generation of correction factors, allowing computation of outdoor PWC for *T*_wb_, Humidex, and Heat Index. The clothing ensembles are shown in (**F**) and (**L**)

The WBGT and UTCI thermal indices account for SOLAR in their calculation, and therefore, a pooled model accounts well for both SHADE and SOLAR data. In both clothing conditions (Fig. 3), changing the model parameters (PWC50 and HillSlope) for the SOLAR data did not improve the fit compared to a pooled model (*p* < 0.05). In other words, the WBGT and UTCI account well for the decrease in PWC with SOLAR, indicated by a rightward shift in their value when SOLAR is added. This was true in both clothing conditions. Consequently, the inclusion of data with SOLAR does not negatively impact the error variance in the model if using WBGT or UTCI to assess the environment, so there is no requirement to adapt the models already presented in our companion paper (table 3 in Foster et al., 2021b). Since the *T*_wb_, Humidex, and Heat Index do not intrinsically account for SOLAR radiation, their values for a certain *T*_a_/RH combination are unchanged with SOLAR, and the inclusion of SOLAR data decreases the predictive capacity of the model substantially. Hence, those heat stress indices require separate models for the SOLAR and SHADE (*p* < 0.05) since less residual variance was documented if separate models were produced for each dataset. This was true in both clothing conditions. For the heat stress indices that cannot use a pooled/global model, the *difference* between the SHADE and SOLAR functions were modeled from a Gaussian expression. Those functions were used to form the correction factors (Table 2), where PWC predictions obtained from SHADE data are adjusted based on the solar load. These equations are based on the red area fill in Fig. 3, which show the *difference* in PWC between SOLAR and SHADE conditions.

**Table 2:** Correction factors (3rd column) for calculation of physical work capacity in outdoor working conditions with exposure to SOLAR at 800 W.m^−2^

Heat metric (°C)	Original PWC equation (Foster et al. 2021)	Correction factor at 800 W.m^−2^ radiation influx	Heat metric range (°C)
***Low clothing coverage (0.26 Clo)***			
WBGT	$\boldsymbol{PWC}=\frac{100}{1+{\left[\frac{33.51}{{WBGT}}\right]}^{-6.76}}$	***Not required***	12–36
UTCI	$\boldsymbol{PWC}=\frac{100}{1+{\left[\frac{45.08}{{UTCI}}\right]}^{-4.78}}$	***Not required***	15–50
*T*_wb_	$\boldsymbol{PWC}=\frac{100}{1+{\left[\frac{30.87}{T_{{wb}}}\right]}^{-6.24}}\hbox{--} {\boldsymbol{PWC}}_{\boldsymbol{corr}}$	$\boldsymbol{PW}{\boldsymbol{C}}_{\mathbf{corr}}\kern0.5em =21.04\times {e}^{\left[-0.5{\left(\frac{T_{wb}-30.51}{8.505}\right)}^2\right]}$	10–33
Humidex	$\boldsymbol{PWC}=\frac{100}{1+{\left[\frac{54.59}{{Humidex}}\right]}^{-4.55}}\hbox{--} {\boldsymbol{PWC}}_{\boldsymbol{corr}}$	$\boldsymbol{PW}{\boldsymbol{C}}_{\mathbf{corr}}\kern0.5em =21.02\times {e}^{\left[-0.5{\left(\frac{Humidex-50.65}{10.54}\right)}^2\right]}$	13–55
Heat Index	$\boldsymbol{PWC}=\frac{100}{1+{\left[\frac{54.28}{{Heat}\ {Index}}\right]}^{-4.37}}-{\boldsymbol{PWC}}_{\boldsymbol{corr}}$	$\boldsymbol{PW}{\boldsymbol{C}}_{\mathbf{corr}}\kern0.5em =21.10\times {e}^{\left[-0.5{\left(\frac{Heat\ Index-49.35}{13.02}\right)}^2\right]}$	14–55
***High clothing coverage (0.86 Clo)***			
WBGT	$\boldsymbol{PWC}=\frac{100}{1+{\left(\frac{33.76}{{WBGT}}\right)}^{-5.87}}$	***Not required***	12–36
UTCI	$\boldsymbol{PWC}=\frac{100}{1+{\left(\frac{45.59}{{UTCI}}\right)}^{-3.98}}$	***Not required***	15–50
*T*_wb_	$\boldsymbol{PWC}=\frac{100}{1+{\left[\frac{31.10}{T_{{wb}}}\right]}^{-5.54}}\hbox{--} {\boldsymbol{PWC}}_{\boldsymbol{corr}}$	$\boldsymbol{PW}{\boldsymbol{C}}_{\mathbf{corr}}\kern0.5em =11.18\times {e}^{\left[-0.5{\left(\frac{T_{wb}-21.67}{9.234}\right)}^2\right]}$	10–33
Humidex	$\boldsymbol{PWC}=\frac{100}{1+{\left[\frac{54.71}{{Humidex}}\right]}^{-3.81}}-{\boldsymbol{PWC}}_{\boldsymbol{corr}}$	$\boldsymbol{PW}{\boldsymbol{C}}_{\mathbf{corr}}\kern0.5em =10.73\times {e}^{\left[-0.5{\left(\frac{Humidex-43.95}{15.99}\right)}^2\right]}$	13–55
Heat Index	$\boldsymbol{PWC}=\frac{100}{1+{\left[\frac{55.79}{{Heat}\ {Index}}\right]}^{-2.68}}-{\boldsymbol{PWC}}_{\boldsymbol{corr}}$	$\boldsymbol{PW}{\boldsymbol{C}}_{\mathbf{corr}}\kern0.5em =11.34\times {e}^{\left[-0.5{\left(\frac{Heat\ Index-44.02}{18.05}\right)}^2\right]}$	14–55

The values need to be subtracted from the shaded values in our companion paper (2nd column; Foster et al., 2021b). For calculations of lower radiation levels, see Eq. (10) in the current paper for full model template and Eq. (17) for an example calculation

These correction factors are for the full SOLAR load used in the experiment. Assuming a linear impact of the SOLAR intensity (based on the heat transfer into the body), for lower radiation levels, lower radiated areas, and different SOLAR angles, the SOLAR impact can be scaled as

16 $${RSF}=\frac{Global\; solar \; intensity\;({W}.{{m}^{-2}})}{800\ {W}.{{m}^{-2}}}\times \frac{{Irradiated}\;{area}\ (\%)}{24.2\%}\times{\sin}({Alpha})$$

where RSF is radiation scale factor (ranging from 0 to 1). Irradiated area can be calculated based on solar altitude and azimuth (Underwood and Ward, 1966). With Alpha=angle (°) between radiation beam and projected surface, i.e., when radiation falls perpendicular onto surface, Alpha=90° and Sin(Alpha)=1.

Example calculation

Below, we provide an example calculation based on the equation template set out in Eq. (10) of the present paper. The example is based on *T*_wb_, in which the correction factor is shown in Table 2. The calculation below assumes a *T*_wb_ of 30°C, a SOLAR intensity of 600 W∙m^−2^, projected over 15% BSA, and the radiation hitting the radiated surface at an angle of 45° (0.785 radians), with low clothing coverage.

17 $$\mathbf{PWC}\%=\frac{100}{1+{\left(\frac{30.98}{{{T}}_{{wb}}}\right)}^{-5.90}}-\left[\frac{600}{800}\times \frac{15}{24.2}\times {\sin}(0.785)\left(21.04\times {{e}}^{\left[-0.5{\left(\frac{30-30.51}{8.505}\right)}^2\right]}\right)\ \right]$$

In this example, PWC is 48%. Without any SOLAR, PWC would be 55%. Note that the original/base model of PWC (the left term to the right of the equal sign) should be taken from our companion paper (table 3 in Foster et al., 2021b).

## Discussion

This study builds on our earlier publication that determined Physical Work Capacity across a wide range of combinations of temperature and humidity (Foster et al., 2021b), but without SOLAR present. The primary aim of the present study was to determine the additional impact of SOLAR on human physical work capacity (PWC), with high and low clothing coverage and across a broad range of temperature and humidity combinations. The secondary aim was to evaluate whether PWC can be predicted outdoors with SOLAR using a variety of heat stress indices and finally whether our previously published functions linking PWC to the climate parameters can be used for both indoor (shade) and outdoor (SOLAR) work settings in their current form.

SOLAR reduced PWC by up to 20%, depending on the temperature, humidity, and clothing condition. We found the impact of SOLAR not to be “additive” (Lloyd and Havenith, 2016), to the temperature, humidity, and clothing effects, but instead to show an interaction effect with these variables. With low clothing coverage in dry heat, SOLAR had a negligible impact on PWC when *T*_a_ ≤ 35°C, but PWC decreased exponentially due to SOLAR when *T*_a_ ≥ 40°C (Fig. 1A). When high clothing coverage was adopted in the same climate types, SOLAR caused a consistent linear decrease in PWC when *T*_a_ ≥35°C (Fig. 1C). In humid conditions, the impact of SOLAR was consistent/additive in that the reduction in PWC caused by SOLAR was similar across all levels of *T*_a_ investigated, in both clothing conditions (Fig. 1B, D). Based on our data (comparing Fig. 2A to 2B), high clothing coverage only seems to protect against reductions in PWC caused by SOLAR when *T*_a_ ≥40°C, with the caveat that more protection from ultraviolet radiation may be required if low clothing coverage is adopted, i.e., with sunscreen. The effect of sunscreen on human thermoregulatory function seems to also depend on the climate type (Connolly and Wilcox, 2000) and the composition of the ointment (Aburto-Corona and Aragón-Vargas, 2016), making any potential interaction difficult to predict at this stage.

In low clothing coverage (exposed skin), PWC was severely affected by SOLAR at 45°C/20% RH, but the effect was substantially less with high clothing coverage in the same environment (Fig. 1). Similar “protective” effects of high clothing coverage during military marching in hot-dry conditions (43.3°C) with SOLAR have been described, evidenced by a substantial decrease in sweat output (Adolph, 1947). The response seems to be explained by the absolute *T*_skin_ with SOLAR, which was greater at 45°C *T*_a_/20% RH in low clothing coverage (37.6 ± 0.3°C) compared with high clothing coverage (36.6 ± 0.1°C). It is likely that the lower *T*_skin_ in high clothing coverage is due to the interruption of the direct radiation to the skin by absorption, which serves to reduce direct dry heat gain from SOLAR (Clark and Cena, 1978; Bröde et al., 2010). However, it is worth noting that the beneficial impact of clothing depends on the color and fabric properties (Nielsen, 1990; Havenith et al., 2006a, 2006b, 2006c; Ioannou et al., 2021b). In dry heat when *T*_a_ ≤35°C, where no effect on PWC of adding SOLAR was observed, the added radiative heat gain was likely compensated for by increased sweat evaporation in low clothing coverage compared with high clothing coverage.

Mean *T*_skin_ was a strong predictor of PWC in the heat, independent of clothing or exposure to SOLAR (Fig. 2 for trial average and Fig. S4 for temporal responses). The temporal responses show that SOLAR causes *T*_skin_ to plateau at a value 2–4°C higher than that in SHADE, while *T*_core_ responses were similar between SOLAR and SHADE. These findings are supported by prior work from our group (Foster et al., 2021b) and others (Ioannou et al., 2017, 2021b; Jay et al., 2019), which implicates *T*_skin_ as the primary determinant of work capacity loss during occupational heat stress, while *T*_core_ seems to be managed by pacing behavior. Mechanistically, a study in mice showed that the strength of the afferent nervous system response is directly proportional to the absolute (not relative) *T*_skin_ during heating (Ran et al., 2016), providing strong mechanistic basis for our observations. Of note, the sensors used to measure *T*_skin_ have a highly conductive skin side and an insulated resin to the external environment. While this largely limits environmental influences on the measurement, we cannot exclude some effect of SOLAR directly impacting the sensor. However, the effect on overall mean *T*_skin_ will be limited due to averaging with sensors not exposed to SOLAR (i.e., at the front of the body).

A fixed cardiovascular strain model was chosen as a proxy for self-paced physical work based on a plethora of field data in which workers can freely adjust their pace in hot climates (Wyndham, 1973; Mairiaux and Malchaire, 1985; Kalkowsky and Kampmann, 2006; Bates and Schneider, 2008; Miller et al., 2011). Discussing data from the South African gold mines (Wyndham, 1973), Vogt et al. (1983) observed that *“while productivity and oxygen consumption fell off with increasing wet-bulb temperature, heart rates remained constant around an average of 130–140 beats∙min*^*−1*^*.”* A heart rate of 130 beats∙min^−1^ was therefore chosen, which also represented an occupational intensity on the border of moderate to heavy physical work, as suggested by the World Health Organization (Andersen, 1978). Data from a follow-up experiment with six 1-h work bouts on a single day show that this heart rate represents a full day limit with participants feeling very fatigued upon cessation (Smallcombe et al., 2019). However, it is possible that, e.g., for shorter periods than a day, a higher limit for the fixed heart rate is possible, and that with higher heart rates (i.e., higher workloads), *T*_core_ and dehydration may become a more relevant predictor of the loss in PWC.

WBGT and UTCI are commonly used heat stress indices in biometeorology, and their values account for any change in mean radiant temperature (Havenith and Fiala, 2015). We show that, based on the change in mean radiant temperature with SOLAR (determined empirically by black globe temperature combined with *T*_a_ and RH), the relative shift in the value of WBGT and UTCI predicts the reduction in PWC caused by SOLAR appropriately. Therefore, assuming that globe temperature is correctly measured (spatially and allowed to equilibrate), the WBGT and UTCI can accurately predict PWC in both shaded and unshaded conditions with a single equation. The validity of models that use WBGT to predict PWC (Dunne et al., 2013; Kjellstrom et al., 2018) is therefore not reduced if also applied to outdoor work settings. In contrast, the *T*_wb_, Humidex, and Heat Index do not intrinsically account for any change in mean radiant temperature, highlighting the importance of context if such indices are linked with human physiology or survival. Correction factors for PWC are provided if such indices are to be used for outdoor work with SOLAR, as shown in Table 2. The equations reported in this study have immediate applicability for those studying the impact of hot weather on PWC, especially in outdoor settings.

It is of interest that WBGT and UTCI work well for the present data which have highly fluctuating metabolic rates. WBGT as an index does not contain a metabolic rate component, as this is only applied when considering the limits to WBGT for different work types (ISO7933, 2004; Havenith and Fiala, 2015). Nevertheless, it seems to reflect the external stress well for variations in temperature, humidity, and SOLAR exposure. It does not work well representing the strain caused by air movement however (Foster et al., 2021a). Conversely, UTCI contains a metabolic rate, i.e., that of a slowly walking person (Bröde et al., 2012). Despite the variable work rate in the experiments in this and the companion papers, UTCI performs well in predicting the strain in relation to temperature, humidity, solar exposure, *and* air movement. The fact that the UTCI metabolic rate is not the same as in the actual conditions does not seem to diminish its predictive power in representing heat strain. Thus, although by design UTCI’s metabolic rate is fixed, it does not seem to limit its application to fixed activity scenarios.

Limitations

There are several limitations of the present study that should be considered. First, this study population was primarily young male participants. The confounding effect of age and sex is pertinent since females and older individuals make up a significant percentage of the heat exposed workforce (varying by industry and country). However, the primary question was the impact of SOLAR, which in and of itself required over 120 empirical trials. To accurately address the confounding effect of individual factors would require an equivalent amount of trials in older and female individuals. Only being able to test in a specific phase of the menstrual cycle would also potentially reduce the number of tests that can be performed in females. Since our recent review suggests that the effect of age and sex are of importance primarily at high heat loads (Foster et al., 2020), it is possible that these populations would show more severe reductions in PWC at the higher ranges of WBGT. We recently demonstrated that fitness level has its largest impact on PWC when WBGT is between 25 and 35°C (Foster et al., 2021c). As fitness has been identified as the main parameter explaining the impact of age (Havenith et al., 1995; Foster et al., 2020), the effect of age may show the same impact range in paced work.

Second, it is unclear if the general conclusions about SOLAR are accurate during exposure to a full working day, in contrast to the 1-h work bout used in our study. Preliminary data from our laboratory (Smallcombe et al., 2019) indicates minimal impact of work duration on PWC until WBGT reaches 36°C, which is only surpassed at 45°C/20% RH and 35°C/80% RH with the addition of SOLAR. Finally, a constant intensity of 800 W∙m^−2^ SOLAR was chosen for the present study. Ideally, different intensities would be measured to improve impact analysis in different regions or times of day. The choice of radiation intensity and radiated surface area represented a worst-case scenario, simulating outdoor work under direct sunlight at the hottest part of the day (Monteith and Unsworth, 1990). The correction factors are thus based on the assumption that the radiation level can be described as a linear impact between our shade and 800 W∙m^−2^ condition. While from a heat balance modeling perspective, this is deemed plausible, research should verify the validity of this assumption.

## Conclusions

Addition of solar radiation to climatic heat stress reduced PWC by up to a further 20%, compared to the same shaded condition, but this is depending on the temperature, humidity, and clothing condition. We observed an interactive effect of solar radiation on physical work capacity, depending on clothing coverage and the climate. WBGT and UTCI account well for the impact of solar radiation on physical work capacity. Correction factors are available if using *T*_wb_, Humidex, or Heat Index for the prediction of physical work capacity, in solar radiation conditions. For choice of climate index in conditions with solar or thermal radiation, WBGT and UTCI are clearly superior.

## Supplementary Information

ESM 1 (PDF 498 kb)
