# Supplementary material for: Quantifying the impact of heat on human physical work capacity; part III: the impact of solar radiation varies with air temperature, humidity, and clothing coverage
Source: Int J Biometeorol. 2021 Oct 28;66(1):175–88. doi: 10.1007/s00484-021-02205-x (PMC8727397; doi:10.1007/s00484-021-02205-x)
Supplement: Supplementary file 1 — (PDF 498 kb) [file 484_2021_2205_MOESM1_ESM.pdf]

Supplementary file

This supplementary file contains:

1. A study design schematic with accompanying description.
2. The trials completed for each individual participant.
3. Individual responses for high and low clothing coverage.
4. Temporal core and skin temperature responses to each condition.

Study schematic

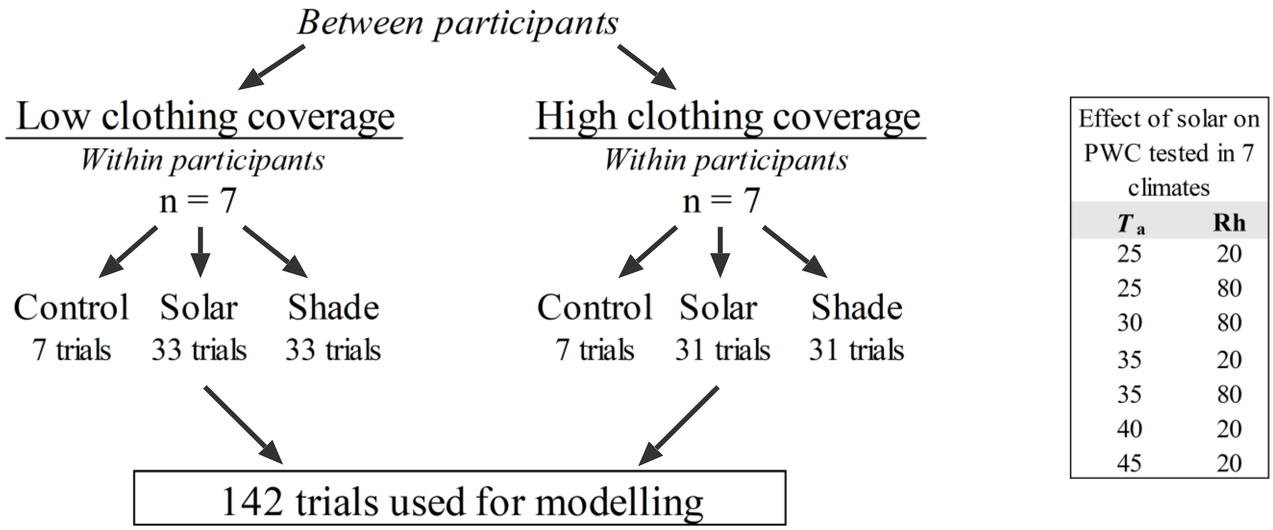

Figure S1. Study schematic. Fourteen participants were enrolled, with 7 completing SOLAR vs SHADE trials in low clothing coverage (0.3 Clo), and 7 in high clothing coverage (0.9 Clo). After completing a trial in a reference (control) environment of 15°C, 50% Rh, SOLAR vs SHADE comparisons were conducted. Experimental trials were always completed on separate days, at the same time of day within subjects. A SOLAR vs SHADE trial was always completed in the same environment sequentially (one followed the other). For example, if a SOLAR trial was conducted at 25°C, 80% Rh, the next trial would be in the same environment, but in SHADE. All data collected in this study was used for modelling purposes (see Figure 3 main text).

Individual trials completed by each participant.

Table S1. Trials performed by each participant. Apart from the reference/control condition, which was completed once, a cross denotes that a participant has completed both a shaded and solar trial within the specified environment.

Low clothing coverage									
		**Participant ID**							
**T_a**	**Rh**	**1**	**3**	**5**	**7**	**8**	**10**	**11**	
15	50	*x*	*x*	*x*	*x*	*x*	*x*	*x*	**7**
25	20	*x*	*x*	*x*	*x*	*x*			**10**
	80	*x*	*x*		*x*	*x*		*x*	**10**
30	80	*x*	*x*	*x*	*x*	*x*	*x*	*x*	**14**
35	20	*x*	*x*	*x*	*x*	*x*			**10**
	80		*x*		*x*		*x*	*x*	**8**
40	20	*x*	*x*		*x*	*x*		*x*	**10**
45	20		*x*					*x*	**4**
**Total trials**		**11**	**15**	**7**	**13**	**11**	**5**	**11**	**∑ = 73**

High clothing coverage									
		**Participant ID**							
**T_a**	**Rh**	**2**	**3**	**4**	**6**	**9**	**12**	**13**	
15	50	*x*	*x*	*x*	*x*	*x*	*x*	*x*	**7**
25	20	*x*	*x*			*x*	*x*		**8**
	80		*x*	*x*	*x*	*x*	*x*	*x*	**12**
30	80		*x*		*x*	*x*	*x*	*x*	**10**
35	20		*x*	*x*		*x*	*x*	*x*	**10**
	80		*x*			*x*			**4**
40	20	*x*	*x*	*x*		*x*	*x*	*x*	**12**
45	20		*x*			*x*		*x*	**6**
**Total trials**		**5**	**15**	**7**	**5**	**15**	**11**	**11**	**∑ = 69**

Individual physical work capacity responses

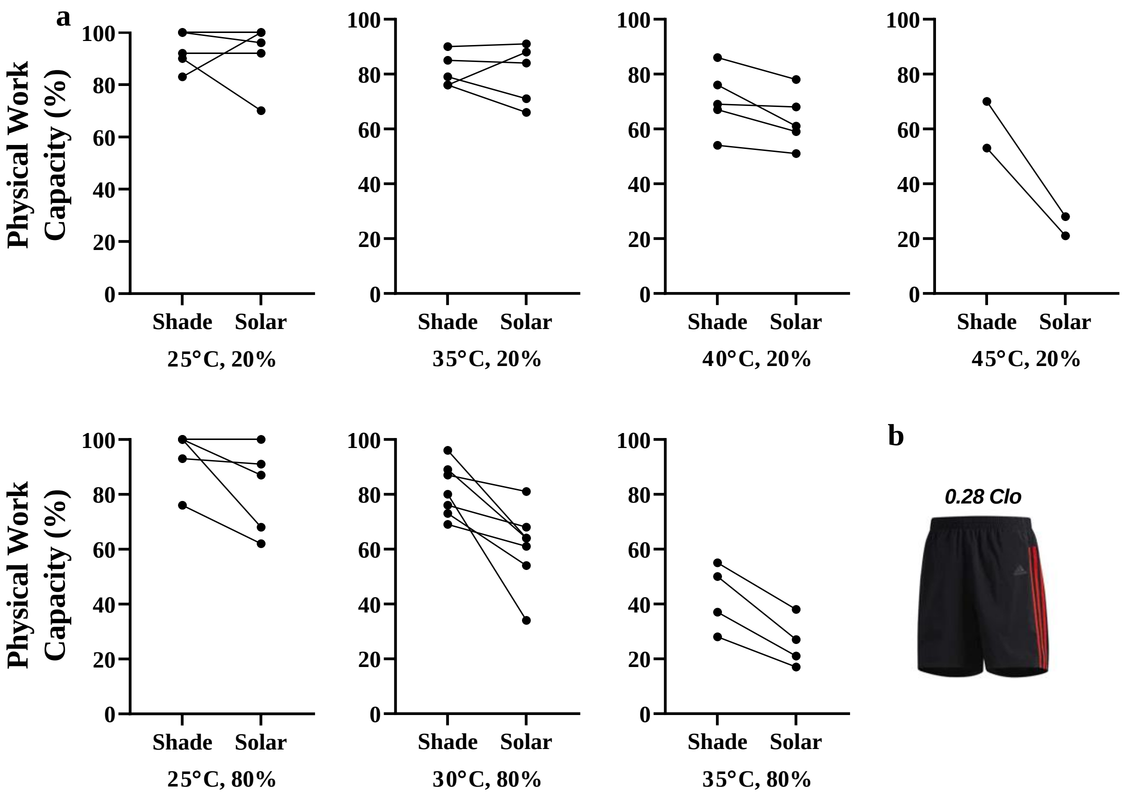

Figure S2. Individual physical work capacity responses in each condition, with and without solar, in low clothing coverage (shown in panel b).

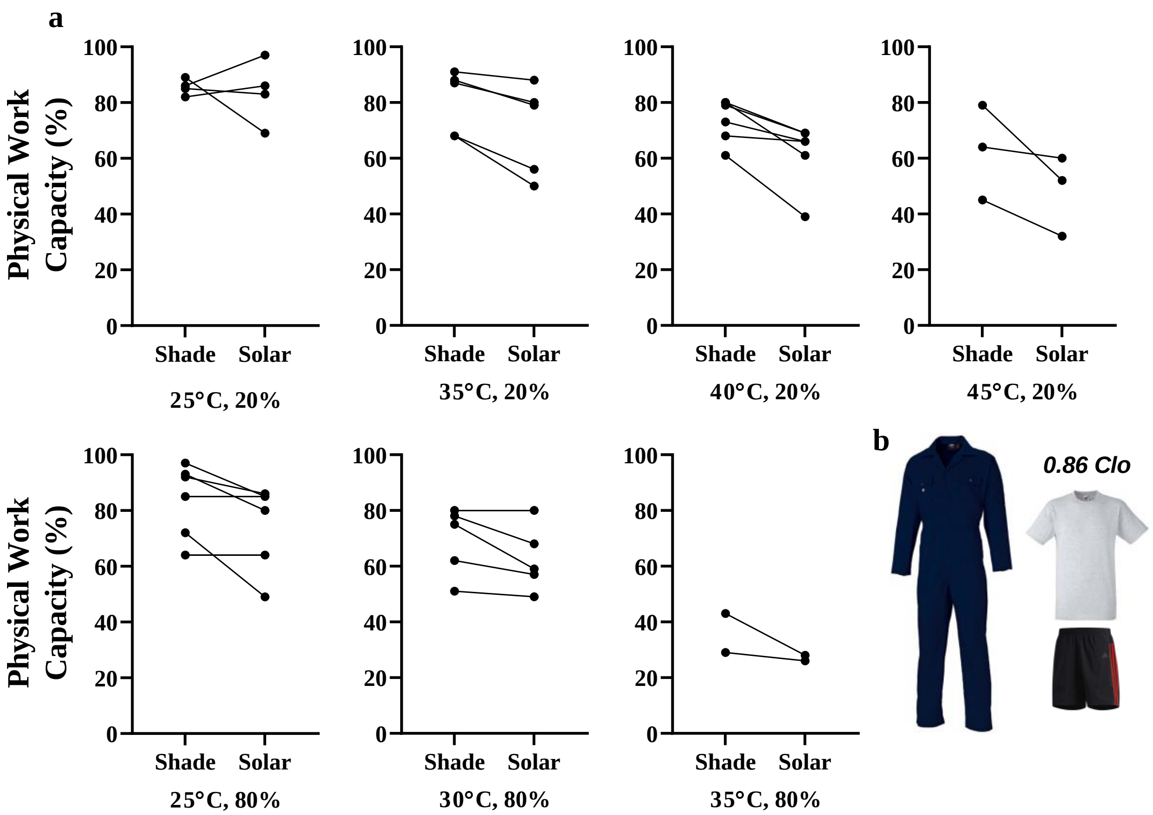

Figure S3. Individual physical work capacity responses in each condition, with and without solar, in high clothing coverage (shown in panel b).

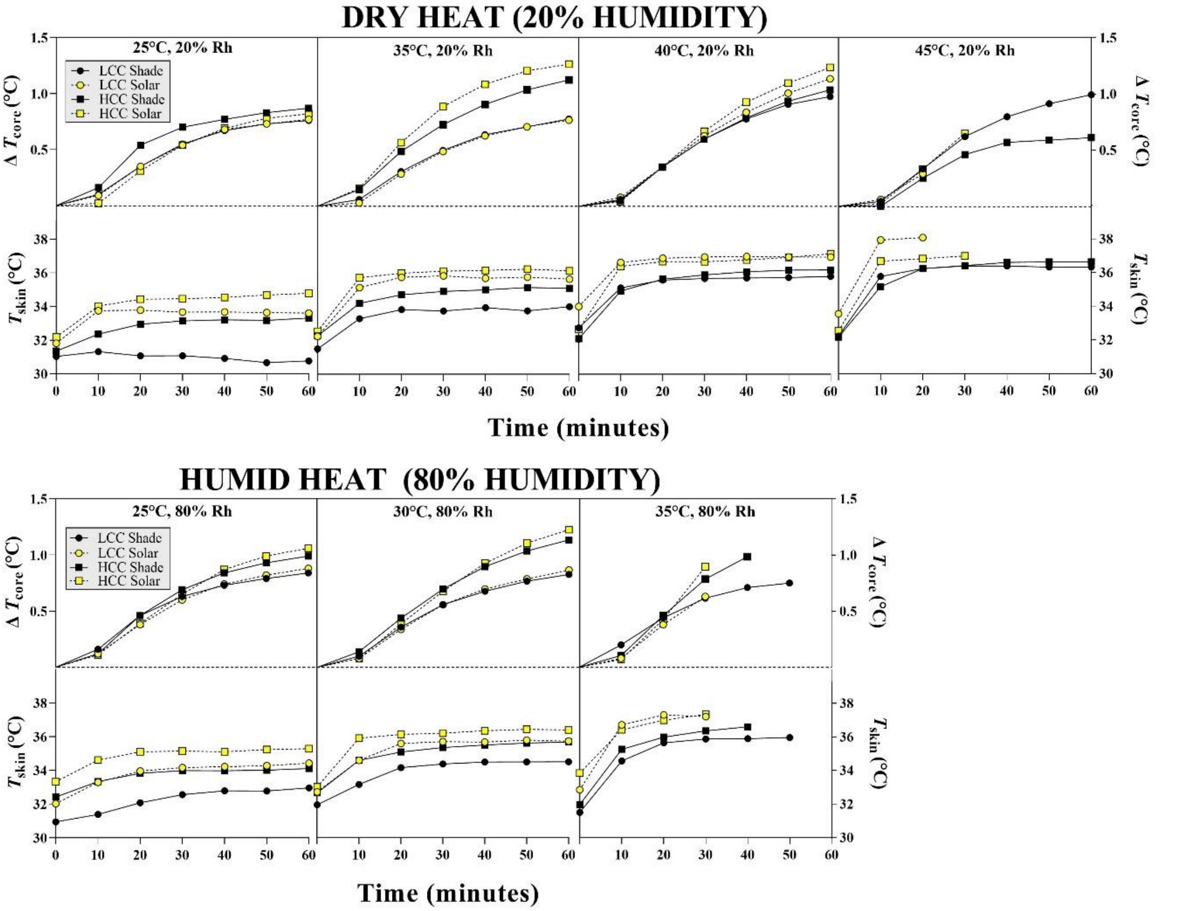

Figure S4. Temporal rectal (core) and mean skin temperature responses. Data are presented as the average score at each time point. Error bars were omitted to increase clarity in data presentation.
